# Bis[μ-4,4′,6,6′-tetra­chloro-2,2′-(piperazine-1,4-diyldimethyl­ene)diphenolato]dicopper(II)

**DOI:** 10.1107/S1600536809049800

**Published:** 2009-11-28

**Authors:** Koji Kubono, Chisato Noshita, Keita Tani, Kunihiko Yokoi

**Affiliations:** a Division of Natural Sciences, Osaka Kyoiku University, Kashiwara, Osaka 582-8582, Japan

## Abstract

In the centrosymmetric dinuclear Cu^II^ title complex, [Cu_2_(C_18_H_16_Cl_4_N_2_O_2_)_2_], the Cu^II^ atom adopts a square-pyramidal geometry with a tetra­dentate ligand in the basal plane. The apical site is occupied by a phenolate O atom from an adjacent ligand, forming a dimer. The mol­ecular structure is stabilized by intra­molecular C—H⋯O and C—H⋯Cl hydrogen bonds.

## Related literature

For the synthesis and the monoclinic and ortho­rhom­bic polymorphs of a tetra­chloro-2,2′-(piperazine-1,4-diyldi­methyl­ene)diphenol, see: Kubono & Yokoi (2007 ▶). For related stuctures, see: Butcher *et al.* (2007 ▶); Kubono *et al.* (2003 ▶); Massoud & Mautner (2004 ▶); Weinberger *et al.* (2000 ▶).
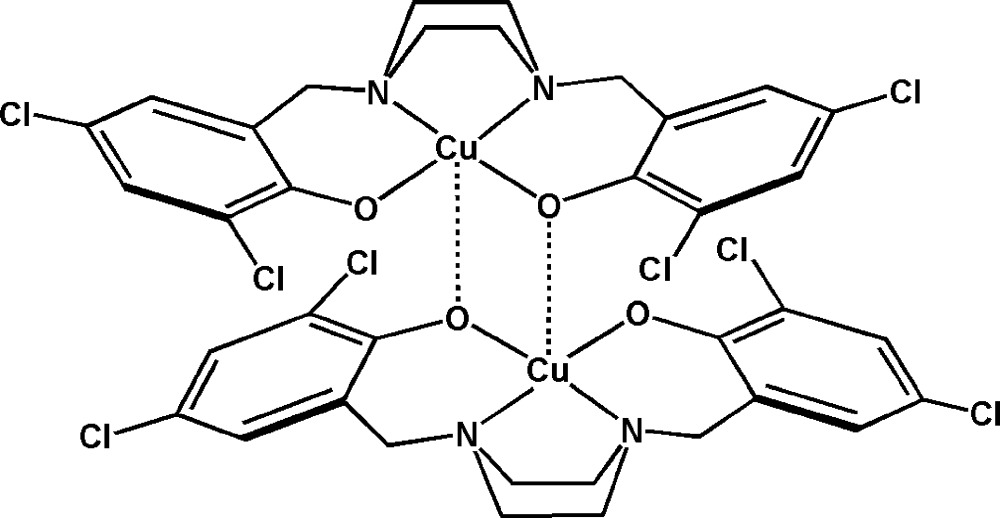


## Experimental

### 

#### Crystal data

[Cu_2_(C_18_H_16_Cl_4_N_2_O_2_)_2_]
*M* *_r_* = 995.36
Monoclinic, 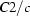

*a* = 20.1772 (18) Å
*b* = 15.3901 (18) Å
*c* = 15.1397 (14) Å
β = 121.140 (6)°
*V* = 4023.9 (7) Å^3^
*Z* = 4
Mo *K*α radiation
μ = 1.63 mm^−1^
*T* = 296 K
0.20 × 0.08 × 0.07 mm

#### Data collection

Rigaku AFC-7R diffractometer
Absorption correction: ψ scan (North *et al.*, 1968 ▶) *T*_min_ = 0.855, *T*_max_ = 0.892
4759 measured reflections
4634 independent reflections
2574 reflections with *I* > 2σ(*I*)
*R*_int_ = 0.046
3 standard reflections every 150 reflections intensity decay: 0.7%

#### Refinement

*R*[*F* ^2^ > 2σ(*F* ^2^)] = 0.041
*wR*(*F* ^2^) = 0.119
*S* = 0.99
4634 reflections
245 parameters
H-atom parameters constrained
Δρ_max_ = 0.37 e Å^−3^
Δρ_min_ = −0.39 e Å^−3^

### 

Data collection: *WinAFC* (Rigaku/MSC, 2006 ▶); cell refinement: *WinAFC*; data reduction: *CrystalStructure* (Rigaku/MSC, 2006 ▶); program(s) used to solve structure: *SIR92* (Altomare *et al.*, 1993 ▶); program(s) used to refine structure: *SHELXL97* (Sheldrick, 2008 ▶); molecular graphics: *PLATON* (Spek, 2009 ▶); software used to prepare material for publication: *CrystalStructure*.

## Supplementary Material

Crystal structure: contains datablocks global, I. DOI: 10.1107/S1600536809049800/fk2007sup1.cif

Structure factors: contains datablocks I. DOI: 10.1107/S1600536809049800/fk2007Isup2.hkl

Additional supplementary materials:  crystallographic information; 3D view; checkCIF report

## Figures and Tables

**Table:** 

Cu1—O1	1.913 (2)
Cu1—O2	1.955 (2)
Cu1—O2^i^	2.381 (3)
Cu1—N1	2.026 (4)
Cu1—N2	2.033 (3)

**Table:** 

O1—Cu1—O2	97.69 (11)
O1—Cu1—O2^i^	96.68 (12)
O1—Cu1—N1	93.97 (12)
O2—Cu1—O2^i^	85.84 (11)
O2—Cu1—N2	92.90 (12)
O2^i^—Cu1—N1	104.18 (13)
O2^i^—Cu1—N2	97.64 (12)
N1—Cu1—N2	73.22 (13)

Symmetry code: (i) 
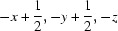
.

**Table 2:** Hydrogen-bond geometry (Å, °)

*D*—H⋯*A*	*D*—H	H⋯*A*	*D*⋯*A*	*D*—H⋯*A*
C11—H12⋯Cl4^i^	0.97	2.76	3.544 (5)	138
C12—H14⋯O1^i^	0.97	2.19	3.112 (6)	159

Symmetry code: (i) 
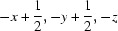
.

**Fig. 1.**
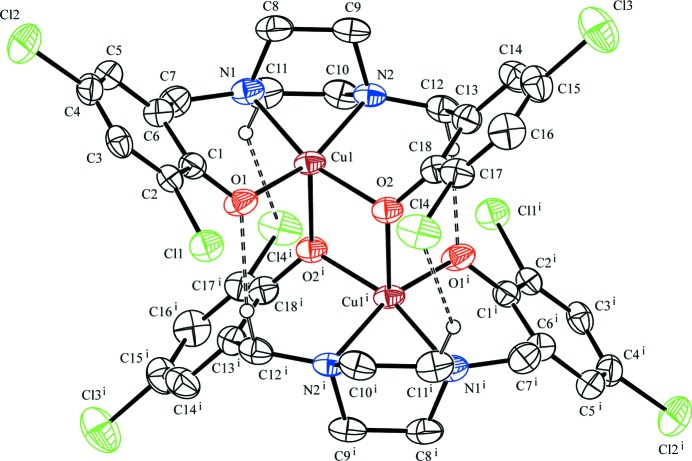
The molecule of the title complex showing the atom-labelling scheme. Displacement ellipsoids are drawn at the 40% probability level. Only the H atoms involved in the hydrogen bonds are represented by circles of arbitrary size. [Symmetry code: (i) 1/2 - x,1/2 - y,-z.]

## supplementary crystallographic information

### Comment

Recently, we have reported the crystal structure of tetrachloro-2,2'-(piperazine-1,4-diyldimethylene)diphenol, H_2_Cl_2_bpi (Kubono & Yokoi, 2007). As a continuation of this work on the structural characterization of piperazinediphenol compounds, the title dinuclear Cu^II^ complex (Fig. 1) is reported here. The Cu^II^ atom has a square-pyramidal coordination geometry with the basal plane comprised of two phenolate O and two tertiary alkyl N atoms from a piperazinediphenol ligand. The apical site is occupied by a phenolate O atom from an adjacent ligand generated by inversion operation, building a centrosymmetric dimer. The Cu···Cu distance within the dimer is 3.1883 (10) Å. The dihedral angle between the benzene rings (C1–C6 and C13–C18) is 87.96 (16) °. The coordination bond lengths and angles (Table 1) are comparable to those observed in related complexes (Butcher *et al.*, 2007; Kubono *et al.*, 2003; Massoud *et al.*, 2004; Weinberger *et al.*, 2000). The molecular structure complex is stabilized by intramolecular C—H···O and C—H···Cl hydrogen bonds (Table 2).

### Experimental

H_2_Cl_2_bpi (0.109 g, 0.25 mmol) was dissolved in 30 ml hot chloroform. Then 30 ml of a methanol solution of copper acetate monohydrate (0.0499 g, 0.25 mmol) were added to this solution. The mixture was stirred for 20 min at 340 K. After a few days at room temperature, dark-green crystals of (I) were obtained. Yield 24.4%. Analysis calculated for C_36_H_32_Cl_8_Cu_2_N_4_O_4_: C 43.44, H 3.24, N 5.63%; found: C 43.05, H 3.22, N 5.53%.

### Refinement

All H atoms were placed at idealized positions and refined as a riding atoms, with C—H = 0.93–0.97Å and *U*_iso_(H) = 1.2 *U*_eq_(C).

### Crystal data

**Table:**

[Cu_2_(C_18_H_16_Cl_4_N_2_O_2_)_2_]	*F*(000) = 2008.00
*M**_r_* = 995.36	*D*_x_ = 1.643 Mg m^−^^3^
Monoclinic, *C*2/*c*	Mo *K*α radiation, λ = 0.71069 Å
Hall symbol: -C 2yc	Cell parameters from 25 reflections
*a* = 20.1772 (18) Å	θ = 13.4–14.9°
*b* = 15.3901 (18) Å	µ = 1.63 mm^−^^1^
*c* = 15.1397 (14) Å	*T* = 296 K
β = 121.140 (6)°	Column, dark-green
*V* = 4023.9 (7) Å^3^	0.20 × 0.08 × 0.07 mm
*Z* = 4	

### Data collection

**Table:**

Rigaku AFC-7R diffractometer	*R*_int_ = 0.046
ω–2θ scans	θ_max_ = 27.5°
Absorption correction: ψ scan (North *et al.*, 1968)	*h* = 0→26
*T*_min_ = 0.855, *T*_max_ = 0.892	*k* = 0→19
4759 measured reflections	*l* = −19→16
4634 independent reflections	3 standard reflections every 150 reflections
2574 reflections with *I* > 2σ(*I*)	intensity decay: 0.7%

### Refinement

**Table:**

Refinement on *F*^2^	H-atom parameters constrained
*R*[*F*^2^ > 2σ(*F*^2^)] = 0.041	*w* = 1/[σ^2^(*F*_o_^2^) + (0.0378*P*)^2^] where *P* = (*F*_o_^2^ + 2*F*_c_^2^)/3
*wR*(*F*^2^) = 0.119	(Δ/σ)_max_ < 0.001
*S* = 0.99	Δρ_max_ = 0.37 e Å^−^^3^
4634 reflections	Δρ_min_ = −0.39 e Å^−^^3^
245 parameters	

### Special details

**Table:**

Geometry. All e.s.d.'s (except the e.s.d. in the dihedral angle between two l.s. planes) are estimated using the full covariance matrix. The cell e.s.d.'s are taken into account individually in the estimation of e.s.d.'s in distances, angles and torsion angles; correlations between e.s.d.'s in cell parameters are only used when they are defined by crystal symmetry. An approximate (isotropic) treatment of cell e.s.d.'s is used for estimating e.s.d.'s involving l.s. planes.
Refinement. Refinement of *F*^2^ against ALL reflections. The weighted *R*-factor *wR* and goodness of fit *S* are based on *F*^2^, conventional *R*-factors *R* are based on *F*, with *F* set to zero for negative *F*^2^. The threshold expression of *F*^2^ > σ(*F*^2^) is used only for calculating *R*-factors(gt) *etc*. and is not relevant to the choice of reflections for refinement. *R*-factors based on *F*^2^ are statistically about twice as large as those based on *F*, and *R*- factors based on ALL data will be even larger.

### Fractional atomic coordinates and isotropic or equivalent isotropic displacement parameters (Å^2^)

**Table:**

	*x*	*y*	*z*	*U*_iso_*/*U*_eq_	
Cu1	0.23572 (3)	0.25394 (3)	0.09411 (4)	0.03688 (13)	
Cl1	0.42437 (6)	0.45428 (7)	0.29614 (8)	0.0506 (2)	
Cl2	0.41605 (8)	0.34818 (11)	0.62836 (9)	0.0812 (4)	
Cl3	−0.07156 (8)	0.53920 (10)	−0.17092 (12)	0.0863 (4)	
Cl4	0.23821 (7)	0.53026 (7)	0.00693 (10)	0.0645 (3)	
O1	0.33424 (16)	0.29710 (19)	0.1996 (2)	0.0490 (7)	
O2	0.21681 (15)	0.33844 (16)	−0.01288 (19)	0.0391 (6)	
N1	0.22348 (19)	0.1741 (2)	0.1911 (2)	0.0421 (7)	
N2	0.12414 (18)	0.2118 (2)	0.0166 (2)	0.0381 (7)	
C1	0.3513 (2)	0.3070 (2)	0.2952 (2)	0.0384 (8)	
C2	0.3933 (2)	0.3796 (2)	0.3535 (2)	0.0356 (8)	
C3	0.4129 (2)	0.3920 (2)	0.4540 (3)	0.0427 (9)	
C4	0.3895 (2)	0.3322 (3)	0.4996 (3)	0.0497 (10)	
C5	0.3496 (2)	0.2591 (2)	0.4474 (3)	0.0498 (10)	
C6	0.3311 (2)	0.2455 (2)	0.3472 (3)	0.0421 (8)	
C7	0.2937 (2)	0.1606 (2)	0.2929 (3)	0.0494 (10)	
C8	0.1593 (2)	0.2145 (3)	0.1957 (3)	0.0550 (11)	
C9	0.0965 (2)	0.2379 (2)	0.0862 (3)	0.0498 (10)	
C10	0.1323 (2)	0.1156 (2)	0.0214 (3)	0.0493 (10)	
C11	0.1958 (2)	0.0924 (2)	0.1308 (3)	0.0509 (10)	
C12	0.0751 (2)	0.2445 (2)	−0.0894 (3)	0.0440 (9)	
C13	0.0784 (2)	0.3422 (2)	−0.0909 (3)	0.0435 (9)	
C14	0.0111 (2)	0.3904 (3)	−0.1272 (3)	0.0548 (11)	
C15	0.0140 (2)	0.4794 (3)	−0.1254 (3)	0.0582 (11)	
C16	0.0832 (2)	0.5220 (3)	−0.0846 (3)	0.0588 (12)	
C17	0.1506 (2)	0.4743 (2)	−0.0473 (3)	0.0474 (9)	
C18	0.1510 (2)	0.3828 (2)	−0.0497 (3)	0.0393 (8)	
H1	0.4417	0.4403	0.4907	0.051*	
H2	0.3350	0.2185	0.4797	0.060*	
H3	0.3308	0.1272	0.2844	0.059*	
H4	0.2805	0.1271	0.3359	0.059*	
H5	0.1772	0.2662	0.2383	0.066*	
H6	0.1391	0.1743	0.2255	0.066*	
H7	0.0488	0.2076	0.0675	0.060*	
H8	0.0865	0.2999	0.0808	0.060*	
H9	0.1462	0.0957	−0.0277	0.059*	
H10	0.0839	0.0884	0.0049	0.059*	
H11	0.1756	0.0535	0.1618	0.061*	
H12	0.2381	0.0634	0.1300	0.061*	
H13	0.0221	0.2259	−0.1168	0.053*	
H14	0.0929	0.2206	−0.1329	0.053*	
H15	−0.0361	0.3622	−0.1529	0.066*	
H16	0.0849	0.5824	−0.0820	0.070*	

### Atomic displacement parameters (Å^2^)

**Table:**

	*U*^11^	*U*^22^	*U*^33^	*U*^12^	*U*^13^	*U*^23^
Cu1	0.0394 (2)	0.0371 (2)	0.0428 (2)	−0.0113 (2)	0.0273 (2)	−0.0080 (2)
Cl1	0.0544 (6)	0.0427 (5)	0.0575 (6)	−0.0089 (4)	0.0308 (5)	−0.0021 (4)
Cl2	0.0865 (9)	0.1165 (12)	0.0375 (6)	0.0042 (8)	0.0298 (6)	−0.0045 (7)
Cl3	0.0662 (8)	0.0757 (9)	0.0988 (11)	0.0224 (7)	0.0297 (7)	−0.0121 (8)
Cl4	0.0670 (7)	0.0423 (6)	0.0935 (9)	−0.0160 (5)	0.0482 (7)	−0.0237 (6)
O1	0.0489 (16)	0.0635 (19)	0.0403 (15)	−0.0248 (14)	0.0272 (13)	−0.0149 (14)
O2	0.0414 (14)	0.0367 (14)	0.0448 (15)	−0.0073 (12)	0.0263 (12)	−0.0059 (12)
N1	0.0474 (19)	0.0402 (19)	0.0470 (19)	−0.0135 (14)	0.0304 (16)	−0.0111 (15)
N2	0.0384 (17)	0.0362 (16)	0.0497 (19)	−0.0090 (14)	0.0298 (16)	−0.0095 (15)
C1	0.0337 (19)	0.045 (2)	0.037 (2)	−0.0012 (16)	0.0183 (16)	−0.0034 (17)
C2	0.0314 (18)	0.0345 (19)	0.038 (2)	0.0022 (15)	0.0159 (16)	−0.0009 (16)
C3	0.037 (2)	0.039 (2)	0.043 (2)	0.0103 (17)	0.0138 (18)	−0.0039 (18)
C4	0.046 (2)	0.064 (2)	0.035 (2)	0.015 (2)	0.0181 (19)	0.003 (2)
C5	0.046 (2)	0.060 (2)	0.046 (2)	0.005 (2)	0.025 (2)	0.011 (2)
C6	0.043 (2)	0.042 (2)	0.045 (2)	−0.0020 (19)	0.0247 (18)	−0.0021 (19)
C7	0.058 (2)	0.040 (2)	0.055 (2)	−0.0046 (19)	0.032 (2)	0.0053 (19)
C8	0.054 (2)	0.068 (3)	0.059 (2)	−0.009 (2)	0.041 (2)	−0.012 (2)
C9	0.049 (2)	0.050 (2)	0.068 (2)	−0.004 (2)	0.043 (2)	−0.011 (2)
C10	0.045 (2)	0.040 (2)	0.066 (2)	−0.0138 (18)	0.031 (2)	−0.016 (2)
C11	0.064 (2)	0.031 (2)	0.066 (2)	−0.0147 (19)	0.039 (2)	−0.0104 (19)
C12	0.038 (2)	0.045 (2)	0.051 (2)	−0.0141 (19)	0.0248 (18)	−0.017 (2)
C13	0.042 (2)	0.046 (2)	0.040 (2)	−0.0026 (18)	0.0205 (18)	−0.0064 (18)
C14	0.040 (2)	0.063 (3)	0.057 (2)	−0.006 (2)	0.022 (2)	−0.014 (2)
C15	0.055 (2)	0.056 (2)	0.055 (2)	0.012 (2)	0.023 (2)	−0.007 (2)
C16	0.072 (3)	0.039 (2)	0.069 (3)	−0.001 (2)	0.038 (2)	−0.009 (2)
C17	0.050 (2)	0.043 (2)	0.055 (2)	−0.0035 (19)	0.031 (2)	−0.011 (2)
C18	0.045 (2)	0.036 (2)	0.040 (2)	−0.0041 (17)	0.0241 (18)	−0.0073 (17)

### Geometric parameters (Å, °)

**Table:**

Cu1—O1	1.913 (2)	C10—C11	1.523 (5)
Cu1—O2	1.955 (2)	C12—C13	1.505 (5)
Cu1—O2^i^	2.381 (3)	C13—C14	1.387 (6)
Cu1—N1	2.026 (4)	C13—C18	1.408 (5)
Cu1—N2	2.033 (3)	C14—C15	1.371 (6)
Cl1—C2	1.740 (4)	C15—C16	1.368 (7)
Cl2—C4	1.751 (4)	C16—C17	1.383 (6)
Cl3—C15	1.752 (5)	C17—C18	1.409 (5)
Cl4—C17	1.744 (4)	C3—H1	0.930
O1—C1	1.313 (5)	C5—H2	0.930
O2—C18	1.332 (4)	C7—H3	0.970
N1—C7	1.472 (4)	C7—H4	0.970
N1—C8	1.470 (7)	C8—H5	0.970
N1—C11	1.484 (5)	C8—H6	0.970
N2—C9	1.479 (7)	C9—H7	0.970
N2—C10	1.487 (4)	C9—H8	0.970
N2—C12	1.472 (4)	C10—H9	0.970
C1—C2	1.406 (4)	C10—H10	0.970
C1—C6	1.419 (6)	C11—H11	0.970
C2—C3	1.373 (6)	C11—H12	0.970
C3—C4	1.372 (7)	C12—H13	0.970
C4—C5	1.373 (5)	C12—H14	0.970
C5—C6	1.378 (6)	C14—H15	0.930
C6—C7	1.519 (5)	C16—H16	0.930
C8—C9	1.521 (5)		
			
O1—Cu1—O2	97.69 (11)	C13—C14—C15	120.3 (4)
O1—Cu1—O2^i^	96.68 (12)	Cl3—C15—C14	119.7 (3)
O1—Cu1—N1	93.97 (12)	Cl3—C15—C16	119.6 (3)
O1—Cu1—N2	162.75 (17)	C14—C15—C16	120.7 (4)
O2—Cu1—O2^i^	85.84 (11)	C15—C16—C17	119.2 (4)
O2—Cu1—N1	163.66 (13)	Cl4—C17—C16	118.3 (3)
O2—Cu1—N2	92.90 (12)	Cl4—C17—C18	119.2 (3)
O2^i^—Cu1—N1	104.18 (13)	C16—C17—C18	122.5 (4)
O2^i^—Cu1—N2	97.64 (12)	O2—C18—C13	122.8 (3)
N1—Cu1—N2	73.22 (13)	O2—C18—C17	121.3 (3)
Cu1—O1—C1	121.8 (3)	C13—C18—C17	115.9 (3)
Cu1—O2—Cu1^i^	94.16 (10)	C2—C3—H1	120.4
Cu1—O2—C18	114.4 (3)	C4—C3—H1	120.4
Cu1^i^—O2—C18	132.1 (2)	C4—C5—H2	119.9
Cu1—N1—C7	115.6 (3)	C6—C5—H2	119.9
Cu1—N1—C8	102.7 (2)	N1—C7—H3	109.1
Cu1—N1—C11	102.5 (2)	N1—C7—H4	109.1
C7—N1—C8	113.9 (3)	C6—C7—H3	109.1
C7—N1—C11	112.1 (2)	C6—C7—H4	109.1
C8—N1—C11	109.0 (3)	H3—C7—H4	107.8
Cu1—N2—C9	102.5 (2)	N1—C8—H5	110.1
Cu1—N2—C10	103.1 (2)	N1—C8—H6	110.1
Cu1—N2—C12	116.0 (2)	C9—C8—H5	110.1
C9—N2—C10	107.9 (3)	C9—C8—H6	110.1
C9—N2—C12	113.3 (3)	H5—C8—H6	108.4
C10—N2—C12	112.8 (3)	N2—C9—H7	110.2
O1—C1—C2	120.9 (4)	N2—C9—H8	110.2
O1—C1—C6	123.2 (3)	C8—C9—H7	110.2
C2—C1—C6	115.8 (3)	C8—C9—H8	110.2
Cl1—C2—C1	117.9 (3)	H7—C9—H8	108.5
Cl1—C2—C3	119.3 (2)	N2—C10—H9	110.3
C1—C2—C3	122.7 (4)	N2—C10—H10	110.3
C2—C3—C4	119.2 (3)	C11—C10—H9	110.3
Cl2—C4—C3	118.7 (3)	C11—C10—H10	110.3
Cl2—C4—C5	120.4 (4)	H9—C10—H10	108.5
C3—C4—C5	120.8 (4)	N1—C11—H11	110.1
C4—C5—C6	120.2 (4)	N1—C11—H12	110.1
C1—C6—C5	121.2 (3)	C10—C11—H11	110.1
C1—C6—C7	118.5 (4)	C10—C11—H12	110.1
C5—C6—C7	120.2 (4)	H11—C11—H12	108.4
N1—C7—C6	112.6 (3)	N2—C12—H13	109.6
N1—C8—C9	107.8 (4)	N2—C12—H14	109.6
N2—C9—C8	107.7 (3)	C13—C12—H13	109.6
N2—C10—C11	107.3 (3)	C13—C12—H14	109.6
N1—C11—C10	107.9 (3)	H13—C12—H14	108.1
N2—C12—C13	110.3 (2)	C13—C14—H15	119.8
C12—C13—C14	119.9 (3)	C15—C14—H15	119.8
C12—C13—C18	118.8 (3)	C15—C16—H16	120.4
C14—C13—C18	121.2 (3)	C17—C16—H16	120.4
			
O1—Cu1—O2—Cu1^i^	96.20 (12)	C8—N1—C7—C6	−66.1 (5)
O1—Cu1—O2—C18	−123.4 (2)	C7—N1—C11—C10	−170.3 (4)
O2—Cu1—O1—C1	134.9 (2)	C11—N1—C7—C6	169.5 (4)
O1—Cu1—O2^i^—Cu1^i^	−97.27 (11)	C8—N1—C11—C10	62.6 (5)
O1—Cu1—O2^i^—C18^i^	31.3 (3)	C11—N1—C8—C9	−63.2 (4)
O2^i^—Cu1—O1—C1	−138.5 (2)	Cu1—N2—C9—C8	−44.0 (3)
O1—Cu1—N1—C7	−12.9 (3)	Cu1—N2—C10—C11	43.3 (4)
O1—Cu1—N1—C8	111.7 (2)	Cu1—N2—C12—C13	−53.1 (4)
O1—Cu1—N1—C11	−135.2 (2)	C9—N2—C10—C11	−64.7 (4)
N1—Cu1—O1—C1	−33.7 (3)	C10—N2—C9—C8	64.4 (3)
O1—Cu1—N2—C9	12.7 (5)	C9—N2—C12—C13	65.1 (4)
O1—Cu1—N2—C10	−99.3 (4)	C12—N2—C9—C8	−169.8 (3)
O1—Cu1—N2—C12	136.8 (4)	C10—N2—C12—C13	−171.8 (4)
N2—Cu1—O1—C1	7.5 (5)	C12—N2—C10—C11	169.2 (4)
O2—Cu1—O2^i^—C18^i^	128.6 (3)	O1—C1—C2—Cl1	−1.8 (5)
O2^i^—Cu1—O2—C18	140.4 (2)	O1—C1—C2—C3	−179.4 (3)
O2—Cu1—N1—C7	−148.4 (3)	O1—C1—C6—C5	−179.4 (3)
O2—Cu1—N1—C8	−23.8 (5)	O1—C1—C6—C7	5.2 (5)
O2—Cu1—N1—C11	89.3 (5)	C2—C1—C6—C5	2.3 (5)
N1—Cu1—O2—Cu1^i^	−128.7 (4)	C2—C1—C6—C7	−173.1 (3)
N1—Cu1—O2—C18	11.7 (5)	C6—C1—C2—Cl1	176.5 (2)
O2—Cu1—N2—C9	−115.2 (2)	C6—C1—C2—C3	−1.0 (5)
O2—Cu1—N2—C10	132.8 (3)	Cl1—C2—C3—C4	−178.9 (3)
O2—Cu1—N2—C12	8.9 (3)	C1—C2—C3—C4	−1.3 (6)
N2—Cu1—O2—Cu1^i^	−97.46 (12)	C2—C3—C4—Cl2	179.2 (3)
N2—Cu1—O2—C18	43.0 (2)	C2—C3—C4—C5	2.5 (6)
O2^i^—Cu1—N1—C7	85.0 (3)	Cl2—C4—C5—C6	−177.9 (3)
O2^i^—Cu1—N1—C8	−150.4 (2)	C3—C4—C5—C6	−1.3 (6)
O2^i^—Cu1—N1—C11	−37.3 (2)	C4—C5—C6—C1	−1.2 (6)
N1—Cu1—O2^i^—Cu1^i^	166.91 (10)	C4—C5—C6—C7	174.1 (4)
N1—Cu1—O2^i^—C18^i^	−64.5 (3)	C1—C6—C7—N1	−55.4 (6)
O2^i^—Cu1—N2—C9	158.6 (2)	C5—C6—C7—N1	129.1 (4)
O2^i^—Cu1—N2—C10	46.6 (3)	N1—C8—C9—N2	−0.5 (4)
O2^i^—Cu1—N2—C12	−77.3 (2)	N2—C10—C11—N1	1.5 (6)
N2—Cu1—O2^i^—Cu1^i^	92.37 (12)	N2—C12—C13—C14	−120.3 (4)
N2—Cu1—O2^i^—C18^i^	−139.0 (3)	N2—C12—C13—C18	56.3 (6)
N1—Cu1—N2—C9	56.1 (2)	C12—C13—C14—C15	177.7 (4)
N1—Cu1—N2—C10	−56.0 (3)	C12—C13—C18—O2	2.5 (7)
N1—Cu1—N2—C12	−179.9 (2)	C12—C13—C18—C17	−176.0 (4)
N2—Cu1—N1—C7	178.8 (3)	C14—C13—C18—O2	179.0 (4)
N2—Cu1—N1—C8	−56.5 (2)	C14—C13—C18—C17	0.5 (7)
N2—Cu1—N1—C11	56.5 (2)	C18—C13—C14—C15	1.1 (8)
Cu1—O1—C1—C2	−140.0 (3)	C13—C14—C15—Cl3	−179.6 (4)
Cu1—O1—C1—C6	41.7 (5)	C13—C14—C15—C16	−2.2 (8)
Cu1—O2—C18—C13	−54.9 (5)	Cl3—C15—C16—C17	179.0 (4)
Cu1—O2—C18—C17	123.5 (4)	C14—C15—C16—C17	1.6 (9)
Cu1^i^—O2—C18—C13	66.2 (6)	C15—C16—C17—Cl4	−178.5 (4)
Cu1^i^—O2—C18—C17	−115.3 (4)	C15—C16—C17—C18	0.2 (6)
Cu1—N1—C7—C6	52.4 (5)	Cl4—C17—C18—O2	−1.0 (7)
Cu1—N1—C8—C9	45.0 (3)	Cl4—C17—C18—C13	177.5 (3)
Cu1—N1—C11—C10	−45.7 (4)	C16—C17—C18—O2	−179.7 (5)
C7—N1—C8—C9	170.8 (3)	C16—C17—C18—C13	−1.2 (7)

Symmetry codes: (i) −*x*+1/2, −*y*+1/2, −*z*.

### Hydrogen-bond geometry (Å, °)

**Table:**

*D*—H···*A*	*D*—H	H···*A*	*D*···*A*	*D*—H···*A*
C11—H12···Cl4^i^	0.97	2.76	3.544 (5)	138
C12—H14···O1^i^	0.97	2.19	3.112 (6)	159

Symmetry codes: (i) −*x*+1/2, −*y*+1/2, −*z*.
